# Are Big Prostates a Challenge? Correlation Between Prostate Volume and Detection Rate in Fusion Biopsy of the Prostate

**DOI:** 10.1155/aiu/5999882

**Published:** 2026-06-14

**Authors:** Toni Franz, Tom Sicker, Julian Lueke, Benny Dinh, Christoph Breininger, Theodore Spinos, Evangelos Liatsikos, Jens-Uwe Stolzenburg

**Affiliations:** ^1^ Department of Urology, University of Leipzig, Liebigstraße 20a, Leipzig, 04103, Germany, uni-leipzig.de; ^2^ Department of Urology, University of Patras, Rio, Patras, 26500, Greece, upatras.gr

**Keywords:** detection rate, fusion biopsy, prostate biopsy, prostate cancer, prostate volume

## Abstract

**Background:**

This study investigates the relationship between prostate cancer (PCa) detection rates and prostate volume. Prostate volume may influence the diagnostic performance of MRI–ultrasound (MRI–US) fusion biopsy. Enlarged prostate glands present specific challenges, including reduced specificity of prostate‐specific antigen (PSA), anatomical alterations of the transition zone, and potential limitations in biopsy accuracy.

**Material and Methods:**

We retrospectively analyzed 1300 consecutive patients with elevated PSA and/or abnormal digital rectal examination (DRE) who underwent MRI–US fusion‐guided biopsy between 2014 and 2023. Patients were stratified into five prostate volume groups (< 30, 30–54.9, 55–79.9, 80–119.9, and ≥ 120 mL). For PI‐RADS–based analyses, three volume categories (< 50, 50–100, and > 100 mL) were applied. Detection rates for overall PCa and clinically significant prostate cancer (csPCa) were assessed.

**Results:**

A total of 1203 patients were included. The overall cancer detection rate was 62.0% (746/1203), with 39.5% (475/1203) classified as clinically significant. Detection rates decreased significantly with increasing prostate volume, from 82.3% in prostates < 30 mL to 28.9% in prostates ≥ 120 mL (*p* < 0.01). Intermediate groups showed detection rates of 67.1%, 50.2%, and 48.5% for volumes of 30–54.9, 55–79.9, and 80–119.9 mL, respectively. A similar trend was observed for csPCa detection (59.4%, 42.3%, 27.4%, 31.5%, and 24.4%; *p* < 0.05). In multivariable analysis, prostate volume remained an independent predictor of cancer detection (OR: 0.99, *p* < 0.01). Stratified by PI‐RADS category, detection rates consistently declined with increasing prostate size. For PI‐RADS 5 lesions, detection decreased from 87.1% in prostates < 50 mL to 83.1% in 50–100 mL and 69.2% in > 100 mL (*p* < 0.01). Comparable trends were observed for PI‐RADS 4 lesions (78.5%, 64.0%, and 50.0%) and PI‐RADS 3 lesions (45.9%, 40.7%, and 31.8%; *p* < 0.05). Additionally, larger prostates were associated with a higher proportion of anterior lesions and a decreasing rate of positive DREs.

**Conclusion:**

Prostate volume is an independent determinant of cancer detection in MRI–US fusion biopsy. Larger prostate glands are associated with significantly reduced detection rates across all PI‐RADS categories. These findings highlight the need to consider prostate volume in diagnostic decision‐making and to adapt biopsy strategies in patients with enlarged prostates.

## 1. Introduction

### 1.1. Current Role of Biopsy and Multiparametric MRI (mpMRI) in Prostate Cancer (PCa) Diagnosis

Histological confirmation by prostate biopsy remains the cornerstone of PCa diagnosis and is mandatory for treatment initiation [1, 2]. However, conventional systematic biopsy is inherently limited, missing up to 40% of clinically significant prostate cancers (csPCas) while simultaneously detecting a substantial proportion of indolent disease [3–6].

mpMRI, interpreted according to the Prostate Imaging Reporting and Data System (PI‐RADS), has fundamentally improved the diagnostic pathway by enabling lesion localization and targeted biopsy [7–13]. In biopsy‐naïve men, mpMRI‐guided biopsy has been shown to increase the detection of clinically significant cancer by approximately 10% compared to systematic biopsy alone [3, 14–17]. Consequently, current guidelines recommend mpMRI as an integral part of primary PCa diagnostics [1, 2].

### 1.2. Limitations of Current Diagnostic Strategies

Despite these advances, neither mpMRI nor targeted biopsy alone provides complete diagnostic accuracy. Both approaches may miss clinically significant tumors, and the combination of targeted and systematic biopsy remains necessary to optimize detection rates [14, 16, 18–21].

These limitations suggest that additional patient‐ or anatomy‐related factors may influence biopsy performance and diagnostic yield.

### 1.3. Diagnostic Challenges in Patients With Enlarged Prostate Volume

One such factor may be prostate volume. Patients with enlarged prostates represent a diagnostically challenging subgroup for several reasons.

First, prostate‐specific antigen (PSA) interpretation is confounded by benign prostatic enlargement, as PSA levels increase proportionally with gland size, reducing specificity for cancer detection [22–24].

Second, structural changes associated with benign prostatic hyperplasia—particularly within the transition zone—may impair biopsy accuracy. Although most PCas arise in the peripheral zone, gland enlargement can compress this region, making it less accessible and more difficult to sample accurately [25–31].

In addition, increasing prostate size may affect technical aspects of biopsy procedures, including targeting precision and sampling efficiency. Attempts to compensate for these challenges, such as saturation biopsy, have not consistently demonstrated improved detection rates [4–6, 31, 32].

### 1.4. Knowledge Gap and Study Aim

While prostate volume is frequently considered in clinical practice, its impact on cancer detection—particularly in the context of MRI/transrectal ultrasound (TRUS) fusion biopsy and across PI‐RADS categories—remains insufficiently characterized. Therefore, the aim of the present study was to evaluate the relationship between prostate volume and PCa detection rates in MRI/TRUS fusion biopsy, both overall and stratified by PI‐RADS category.

We hypothesized that increasing prostate volume is associated with reduced detection rates, even in the presence of suspicious MRI findings.

## 2. Materials and Methods

We retrospectively analyzed 1300 consecutive patients with elevated PSA levels and/or abnormal digital rectal examination (DRE) who underwent MRI–ultrasound (MRI–US) fusion‐guided prostate biopsy at our institution between August 2014 and December 2023. All patients provided written informed consent prior to inclusion. The diagnostic workup included DRE, serum PSA testing, and mpMRI, followed by MRI–US fusion biopsy. Patients with prior definitive prostate treatments (including radiation therapy, brachytherapy, cryotherapy, high‐intensity focused ultrasound, or focal laser ablation) as well as those with incomplete data on PI‐RADS scoring or prostate volume were excluded from the analysis. The requirement for ethical approval was waived by the Ethics Committee of the Medical Faculty of the University of Leipzig (reference number: 280/25‐ek).

### 2.1. mpMRI

mpMRI examinations were performed in several radiology centers using 1.5‐T or 3‐T scanners with phased‐array pelvic coils. The use of an endorectal coil was optional and followed local practice. Despite minor protocol adjustments over time, all examinations adhered to standardized PI‐RADS–compliant imaging protocols. The protocol included T2‐weighted, diffusion‐weighted (DWI), and dynamic contrast‐enhanced (DCE) sequences following intravenous administration of a gadolinium‐based contrast agent (Table 1). To reduce bowel motion artifacts, patients received either butylscopolamine or glucagon prior to imaging. All mpMRI scans were interpreted by board‐certified radiologists with extensive experience in prostate imaging in accordance with PI‐RADS Version 2.1. Image interpretation was centralized. Lesions were scored according to PI‐RADS criteria, applying zone‐specific dominant sequences (DWI for the peripheral zone and T2‐weighted imaging for the transition zone). In equivocal cases (PI‐RADS 3), DCE imaging was used for further characterization. Lesions were delineated on T2‐weighted images using the institutional PACS and assigned to predefined prostate sectors according to PI‐RADS v2.1. Additionally, lesions were categorized according to anatomical location (anterior fibromuscular stroma [AFMS], anterior, or posterior) and zonal position (base, midgland, and apex).

**TABLE 1:** Imaging parameters of multiparametric prostate MRI.

	**Sequence**	**Slices**	**ST (mm)**	**IPR (mm)**	**TR (ms)**	**TE (ms)**	**FOV (mm)**	**FA (°)**

T2w (tra, cor, sag)	TSE	25–26	3.0.	0.4–0.7	4490	101–108	220 × 220	120
DWI (tra)	EPI	20	3.0	1.6 × 1.6	3000	58	220 × 220	90
T1w DCE (tra)	GRASP	24	3.0	1.1 × 1.1	4.0	1.86	240 × 240	12

*Note:* SPGR, spoiled gradient echo; TE, echo time; TR, repetition time.

Abbreviations: DCE, dynamic contrast‐enhanced; DWI, diffusion‐weighted imaging; FA, flip angle; FOV, field of view; IPR, in‐plane resolution; SS‐EPI, single‐shot echo planar imaging; ST, slice thickness; TSE, turbo spin echo.

Prostate and lesion dimensions were measured in three orthogonal planes, and volumes were calculated using the ellipsoid formula (height × width × length × *π*/6). All patients underwent mpMRI prior to biopsy and presented with PI‐RADS scores of 3, 4, or 5. The indication for biopsy was based on PSA levels, DRE findings, life expectancy, comorbidities, and validated risk calculators. MRI findings were not used as an independent determinant for biopsy indication.

### 2.2. Fusion Biopsy and Pathological Analysis

All biopsies were performed via a transrectal approach under local anesthesia in an outpatient setting. Patients were positioned either in lithotomy or left lateral position depending on operator preference.

MRI–US fusion biopsy was performed using the Koelis platform. Targeted biopsies were obtained from MRI‐visible lesions, with 2‐3 cores per lesion, followed by a standard 12‐core systematic biopsy.

Biopsy samples were analyzed individually by specialized uropathologists. In cases of PCa, diagnosis and grading were confirmed by a second board‐certified pathologist. Tumors were classified according to the International Society of Urological Pathology (ISUP) grading system.

csPCa was defined as Gleason score ≥ 3 + 4 (ISUP ≥ 2), PSA ≥ 10 ng/mL, or clinical stage ≥ cT2 [33, 34].

### 2.3. Measurements and Statistical Analysis

Clinical, radiological, and histopathological data were recorded and analyzed with respect to prostate volume, PI‐RADS category, and cancer detection rates. For primary analyses, patients were stratified into five prostate volume groups: < 30, 30–54.9, 55–79.9 mL, 80–119.9 mL, and ≥ 120 mL. This categorization was performed to allow a clinically intuitive comparison of cancer detection rates across different prostate size groups. In addition, prostate volume was also analyzed as a continuous variable in logistic regression models to assess its independent association with PCa detection. To improve interpretability and ensure adequate subgroup sizes—particularly in PI‐RADS–stratified analyses—a secondary analysis using three aggregated volume groups (< 50 mL, 50–100 mL, and > 100 mL) was performed. This approach reflects commonly used clinical volume strata and served as a sensitivity analysis to confirm the robustness of the results. In addition, prostate volume was analyzed as a continuous variable in logistic regression models to assess its independent association with PCa detection. Descriptive statistics were used to summarize patient characteristics. Continuous variables are presented as mean ± standard deviation (SD), and categorical variables are presented as frequencies and percentages. All statistical tests were two‐sided, with *p* < 0.05 considered statistically significant. Univariable and multivariable logistic regression analyses were performed to identify predictors of PCa and csPCa detection. Statistical analyses were conducted using Microsoft Excel and DATAtab (Seiersberg, Austria).

## 3. Results

Between August 2014 and December 2023, a total of 1300 patients who underwent MRI–US fusion‐guided prostate biopsy were identified. Of these, 97 biopsy sessions were excluded due to prior definitive prostate treatment (*n* = 53) or incomplete data (*n* = 44), resulting in a final cohort of 1203 patients.

**FIGURE 1:**
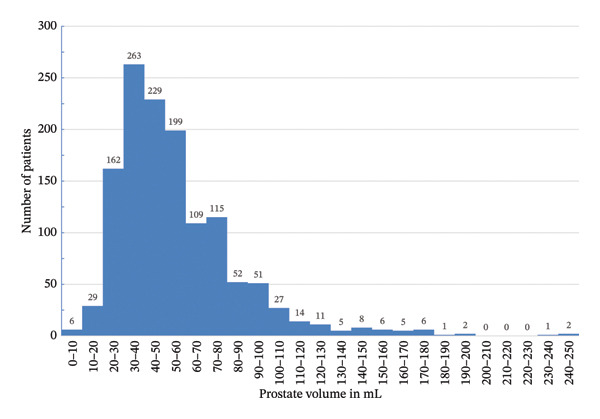
Distribution of prostate volumes in the study cohort (*n* = 1203). The histogram illustrates the frequency of the patients across different prostate volume ranges.

In this cohort, the median age of the patients was 65.32 years, reflecting a typical demographic for PCa evaluation. The average PSA level across the group was 9.13 ng/mL. The mean prostate volume among the patients was calculated at 56.15 mL (mL), with a broad distribution of prostate sizes within the study population (Figure 1). The data, including patient characteristics, lesion characteristics, and detection rates, are presented in Table 2.

**TABLE 2:** Characteristics of patients.

Characteristics	All (1203 patients)	Standard deviation
Age in years	65.3	57.8–72.8
PSA level in ng/mL	9.13	1.29–16.97
PSA density in ng/mL^2^	0.19	0.001–0.38
Prostate volume in cm^3^	56.15	25.89–86.41
< 30 mL	175 (14.55%)–14 pat. with endorectal coil (8%)	
30–54.9 mL	568 (47.2%)–29 pat. with endorectal coil (5.1%)	
55–79.9 mL	285 (23.7%)–17 pat. with endorectal coil (6.0%)	
80–119.9 mL	130 (10.8)–5 pat. with endorectal coil (3.8%)	
> 120 mL	45 (3.75%)–1 pat. with endorectal coil (2.2%)	
Biopsy‐naïve	682/1203 (56.7%)	
Previous biopsies	0.82	0–1.05
Positive DRE		
Positive	144 (12%)	
Negative	1017 (84.5%)	
n/a	42 (3.5%)	
Lesion size in mm	10.12	7.1–13.14
PI‐RADS category		
3	906 (45.25%)–26 pat. with endorectal coil (2.9%)	
4	853 (42.6%)–29 pat. with endorectal coil (3.4%)	
5	243 (12.15%)–11 pat. with endorectal coil (4.5%)	
Use of endorectal coil	66 (5.5%)	
Diagnosis of all PCa	62.01% (746/1023)	
Diagnosis of csPCa	39.5% (475/1023)	

Analyzing biopsy history, 682 of the 1203 patients (56.7%) had never undergone a prior prostate biopsy, whereas 521 patients (43.3%) had previously undergone one or more TRUS‐guided prostate biopsies, averaging 0.82.

The overall detection rate was 62.0% (746/1203). csPCa was detected in 39.5% of all patients (475/1203) and in 63.7% of cancer cases (475/746) (Table 3). Detection rates increased with higher PI‐RADS categories. For PI‐RADS 3, 4, and 5 lesions, detection rates for any prostate cancer (allPCa) were 39.8%, 63.1%, and 80.2%, respectively, while csPCa detection rates were 16.1%, 38.7%, and 67.1%.

**TABLE 3:** Detection rates—PI‐RADS‐independent.

Prostate volume	Overall PCa detection in %	csPCa detection in %
All	62.0	39.5
< 30 mL	82.3	59.4
30–54.9 mL	67.1	42.3
55–79.9 mL	50.3	27.4
80–119.9 mL	48.4	31.5
≥ 120 mL	28.9	33.3

Logistic regression analysis identified several predictors of PCa detection. In univariate analysis, increasing age, higher PSA density, smaller prostate volume, and a positive DRE were significantly associated with the presence of PCa. Prostate volume showed a significant inverse association with cancer detection (OR: 0.98, 95% CI: 0.98–0.99, *p* < 0.001), whereas PSA density demonstrated the strongest positive association (OR: 29.43, 95% CI: 10.83–80.00, *p* < 0.001). Serum PSA showed only a weak association (OR: 1.02, 95% CI: 1.00–1.04, *p* = 0.014), and prior biopsy status was not associated with cancer detection.

In multivariable analysis, prostate volume remained an independent predictor of PCa detection (OR: 0.99, 95% CI: 0.98–0.996, *p* = 0.002), alongside age, PSA density, and positive DRE findings. PSA density remained the strongest predictor (OR: 27.97, 95% CI: 3.57–219.38, *p* = 0.002), while serum PSA and prior biopsy were not independently associated with cancer detection (Table 4).

**TABLE 4:** Association between overall prostate cancer (all PCA) and other clinical factors—left column: univariate analysis; right column: multivariate analysis.

Variables	Univariable analysis vs. all PCa odds ratio (95% CI), *p* value	Multivariable analysis vs. all PCa odds ratio (95% CI), *p* value
Age in years	1.05 (1.04–1.07), *p* < 0.001	1.06 (1.04–1.08), *p* = 0.004
Previous biopsies	1.00 (0.90–1.11), *p* = 0.954	0.96 (0.85–1.08), *p* = 0.471
Prostate volume in mL	0.96 (0.94–0.99), *p* < 0.001	0.99 (0.98–1.00), *p* = 0.002
Serum‐PSA in ng/mL	1.88 (1.69–2.08), *p* < 0.011	0.97 (0.98–1.01), *p* = 0.191
PSA density in ng/mL/cm^3^	22.35 (17.25–31.85), *p* < 0.001	27.97 (3.57–99.2), *p* = 0.002
Size of the lesion in mm	1.07 (1.05–1.08), *p* = 0.045	1.11 (0.99–1.15), *p* = 0.061

For csPCa, similar associations were observed. In multivariable analysis, age (OR: 1.07, 95% CI: 1.05–1.09, *p* < 0.001), prior biopsy (OR: 0.72, 95% CI: 0.62–0.83, *p* < 0.001), prostate volume (OR: 0.99, 95% CI: 0.98–1.00, *p* = 0.003), and PSA density (OR: 17.64, 95% CI: 2.65–117.36, *p* = 0.003) remained independent predictors (Table 5).

**TABLE 5:** Association between clinically significant prostate cancer (csPCa) and other clinical factors—left column: univariate analysis; right column: multivariate analysis.

Variables	Univariable analysis vs. csPCa odds ratio (95% CI), *p* value	Multivariable analysis vs. csPCa odds ratio (95% CI), *p* value
Age in years	1.06 (1.04–1.08), *p* < 0.001	1.07 (1.05–1.09), *p* < 0.001
Previous biopsies	0.85 (0.75–0.96), *p* = 0.01	0.72 (0.62–0.83), *p* < 0.001
Prostate volume in mL	0.98 (0.98–0.99), *p* < 0.001	0.98 (0.98–1.00), *p* = 0.003
Serum‐PSA in ng/mL	2.04 (1.84–2.86), *p* < 0.001	1.02 (0.97–1.06), *p* = 0.472
PSA density in ng/mL/cm^3^	35.54 (15.13–83.51), *p* < 0.001	17.64 (2.65–81.37), *p* = 0.003
Size of the lesion in mm	1.09 (0.99–1.17), *p* = 0.12	1.03 (0.99–1.08), *p* = 0.15

**FIGURE 2:**
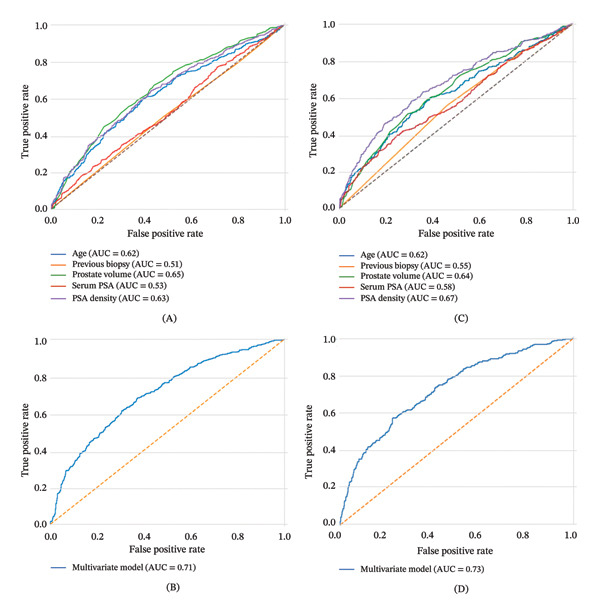
Receiver operating characteristic (ROC) curves illustrating the diagnostic performance of clinical parameters for the prediction of overall prostate cancer (allPCa) and clinically significant prostate cancer (csPCa). (A, B) allPCa: (A) Univariate logistic regression models including age, prior biopsy status, prostate volume, PSA, PSA density, and DRE. Prostate volume showed the highest discriminative ability (AUC = 0.65), followed by PSA density (0.63) and age (0.62), while PSA (0.53) and prior biopsy (0.51) showed limited performance. (B) Multivariable model including all parameters (AUC = 0.71). (C, D) csPCa: (C) Univariate models demonstrating PSA density as the strongest predictor. (D) Multivariable model with improved diagnostic performance (AUC = 0.73). The dashed diagonal line represents random classification (AUC = 0.5).

The discriminative performance of the models was evaluated using receiver operating characteristic (ROC) analysis. Prostate volume showed moderate predictive ability (AUC: 0.648), comparable to PSA density (AUC: 0.634) and age (AUC: 0.617), while serum PSA and prior biopsy showed limited performance. The combined multivariable model achieved an AUC of 0.718, indicating moderate diagnostic accuracy (Figure 2).

**FIGURE 3:**
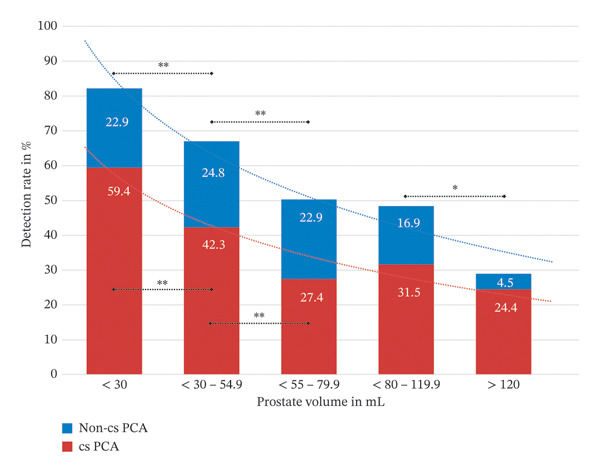
Prostate cancer detection rate and prostate volume; classification into clinically significant (cs—red) and clinically insignificant (non‐cs—green) cancers; dotted black line indicates statistical significance; ^∗^ *p* < 0.05; ^∗∗^ *p* < 0.01.

Detection rates decreased with increasing prostate volume. The overall detection rate was highest in prostates < 30 mL (82.3%) and declined to 67.1%, 50.2%, 48.5%, and 28.9% for prostate volumes of 30–54.9, 55–79.9, 80–119.9, and ≥ 120 mL, respectively. A similar trend was observed for csPCa detection (59.4%, 42.3%, 27.4%, 31.5%, and 24.4%, respectively). Significant differences were observed between the groups < 30 and 30–54.9 mL (*p* < 0.01), 30–54.9 and 55–79.9 mL (*p* < 0.01), and 80–119.9 and ≥ 120 mL (*p* < 0.05) (Table 3, Figure 3).

Lesion location varied across prostate volume groups, with an increasing proportion of anterior lesions observed in larger prostates. In prostates ≥ 120 mL, 70.0% of lesions were located anteriorly, whereas posterior lesions accounted for 27.5% (Table 4). AFMS lesions remained infrequent across all volume groups (Table 6).

**TABLE 6:** Detection rates of overall prostate cancer stratified by prostate volume and lesion location within the prostate.

(mL)	AFMS	Anterior	Posterior
< 30	12 (5.7%)	91 (43.1%)	108 (51.2%)
30–54.9	50 (6.0%)	415 (50.1%)	364 (43.9%)
55–79.9	27 (3.7%)	379 (51.6%)	329 (44.8%)
80–119.9	10 (3.5%)	148 (51.7%)	128 (44.8)
> 120	2 (2.5%)	56 (70.0%)	22 (27.5%)

Furthermore, the proportion of patients with a positive DRE decreased with increasing prostate volume, from 18.5% in prostates < 30 mL to 4.0% in prostates ≥ 120 mL (Table 5). Among DRE‐positive patients, biopsy positivity for PCa remained high across all volume groups, whereas rates for csPCa showed variability, particularly in larger prostates (Table 7).

**TABLE 7:** Detection rates of overall prostate cancer (allPCa) and clinically significant prostate cancer (csPCa) stratified by prostate volume in patients with a positive digital rectal examination (DRE).

(mL)	DRE‐positive *n* (%)	All PCA in DRE+ (%)	cs PCA in DRE+ (%)
< 30	30 (18.5%)	29 (96.7%)	21 (70.0%)
30–54.9	73 (12.5%)	66 (90.4%)	40 (54.8%)
55–79.9	28 (8.2%)	26 (83.9%)	19 67.9%)
80–119.9	11 (6.6%)	8 (72.7%)	5 (45.5%)
> 120	2 (4.0%)	2 (100%)	2 (100%)

**FIGURE 4:**
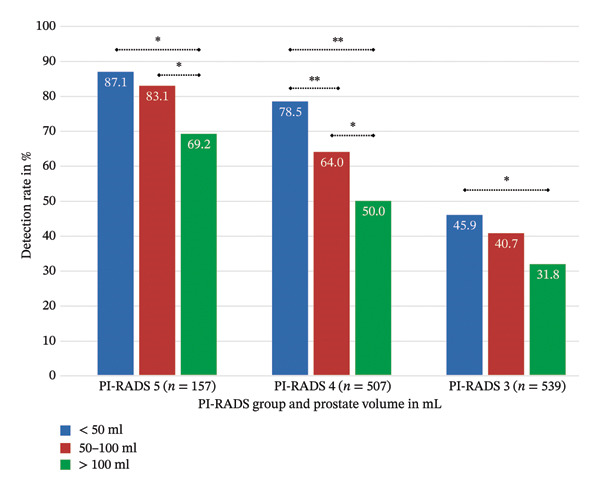
Prostate cancer detection rate stratified by PI‐RADS score and prostate volume; dotted black line indicates statistical significance; ^∗^ *p* < 0.05; ^∗∗^ *p* < 0.01^∗^.

When stratifying by both PI‐RADS category and prostate volume, detection rates decreased with increasing prostate size across all PI‐RADS groups. For PI‐RADS 5 lesions, detection rates declined from 87.1% in prostates < 50 mL to 83.1% in 50–100 mL and 69.2% in > 100 mL (*p* < 0.01). Similar trends were observed for PI‐RADS 4 lesions (78.5%, 64.0%, and 50.0%; *p* < 0.01 and *p* < 0.05) and PI‐RADS 3 lesions (45.9%, 40.7%, and 31.8%; *p* < 0.01) (Table 8, Figure 4).

**TABLE 8:** Detection rates of overall prostate cancer stratified by prostate volume and PI‐RADS score (in %).

(mL)	PI‐RADS 3	PI‐RADS 4	PI‐RADS 5
< 50	45.9	78.5	87.1
50–100	40.7	64.0	83.1
≥ 100	31.8	50.0	69.2

**FIGURE 5:**
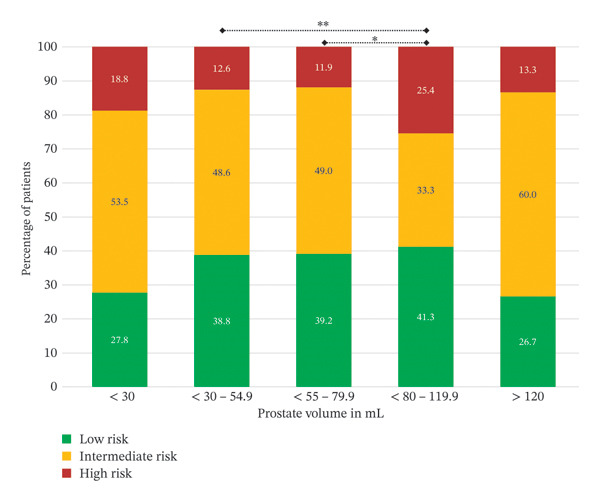
Cancer grading stratified by prostate volume; low risk: green; intermediate risk: yellow; high risk: red; dotted black line indicates statistical significance; ^∗^ *p* < 0.05; ^∗∗^ *p* < 0.01.

In the analysis of tumor aggressiveness, a higher proportion of high‐risk cancers was observed in patients with prostate volumes of 80–119.9 mL compared to those with volumes of 30–54.9 and 55–79.9 mL (25.4% vs. 12.6% and 11.9%, respectively; *p* < 0.05). No consistent trend across all prostate volume groups was observed, and no statistically significant differences were found between the remaining groups (Figure 5).

## 4. Discussion

### 4.1. Limitations of Current Biopsy Techniques in PCa Diagnosis

Despite substantial advances in imaging, no modality currently provides sufficient specificity to replace histopathological confirmation in PCa diagnostics. Tissue sampling therefore remains mandatory prior to treatment initiation. However, due to the anatomical location of the prostate within the pelvis, uniform and representative tissue sampling remains challenging [31].

Conventional TRUS‐guided systematic biopsy is limited by its inability to reliably detect small or isoechoic lesions. Reported detection rates range between 27% and 40% [4–6, 35], and hypoechoic lesions are malignant in only 17%–57% of cases, whereas up to 56% of cancers arise in isoechoic regions [36].

In addition, technical limitations of MRI–US fusion biopsy may further affect detection accuracy. In particular, increasing prostate volume may impair fusion precision due to deformation effects during probe manipulation [35]. Furthermore, the optional use of an endorectal coil during mpMRI can alter prostate shape, potentially leading to misregistration during fusion and reduced targeting accuracy [37].

### 4.2. Strategies to Improve Biopsy Accuracy

Various strategies have been proposed to improve biopsy quality, including saturation biopsy with an increased number of cores [4–6, 38], additional anterior horn biopsies [39–45], contrast‐enhanced ultrasound (CEUS), or systematic grid‐guided transperineal biopsy. However, the clinical benefit of these approaches remains controversial [3, 46].

Eskicorapci et al. reported that adding four lateral peripheral biopsies to the conventional 10‐core biopsy increased cancer detection by 25.5% without significantly increasing morbidity or the detection of clinically insignificant cancers [44]. Descazeaud et al. similarly reported improved accuracy in predicting pT3 tumors using saturation biopsy compared with standard biopsy protocols [6].

Other authors, however, suggest that the main benefit of saturation biopsy lies in the context of active surveillance, repeat biopsy, or targeted sampling of the anterior horn [4, 40, 47, 48]. Jones et al. found no advantage of saturation biopsy as an initial diagnostic strategy. Although saturation biopsy may increase cancer detection after a previous negative biopsy, it does not appear to offer a benefit as a primary biopsy approach [5].

Eichler et al. reached a similar conclusion in their analysis of 87 studies including more than 20,000 patients. While increasing the number of biopsy cores was associated with higher cancer detection, a protocol of 12 cores including laterally directed biopsies provided the best balance between detection rates and adverse events [49]. More individualized approaches have therefore been proposed, including adjusting biopsy strategies to prostate volume [42]. This concept is particularly relevant in the context of MRI‐targeted biopsy, where anatomical and volumetric factors may influence targeting accuracy.

### 4.3. Diagnostic Challenges in Patients With Enlarged Prostate Volume

Patients with enlarged prostates and suspected malignancy present particular diagnostic challenges. First, PSA levels are more difficult to interpret in this population, as benign prostatic enlargement can also lead to increased serum PSA levels independent of malignancy [22–24]. PSA levels rise proportionally with prostate size, meaning that elevated values do not necessarily indicate malignancy.

Second, enlargement of the transition zone may compromise biopsy accuracy. Although the majority of PCas arise in the peripheral zone, gland enlargement may compress this region, reducing accessibility and sampling precision [25–31]. While our findings support the hypothesis that anatomical changes in enlarged prostates may impair cancer detection, we did not specifically assess the relative size of the transition zone in relation to the peripheral zone. The TZ:PZ ratio may represent a relevant parameter to further investigate the potential impact of peripheral zone compression on the detection of csPCa and should be addressed in future studies.

Targeted biopsies of suspicious lesions identified by imaging may therefore represent an important strategy to improve detection. mpMRI has become a valuable tool for identifying, localizing, and guiding biopsies of suspicious lesions [9, 10, 13, 16, 50–52].

### 4.4. Impact of Prostate Volume on Cancer Detection in the Present Study

In the present study, prostate volume emerged as a strong and independent determinant of cancer detection. Detection rates declined substantially with increasing gland size, both for overall PCa and clinically significant disease.

Specifically, overall detection rates decreased from 82.3% in prostates < 30 mL to 28.9% in prostates ≥ 120 mL (*p* < 0.01). Importantly, this inverse association remained significant in multivariable analysis, confirming prostate volume as an independent predictor of cancer detection.

Notably, this effect persisted across all PI‐RADS categories. Even in highly suspicious lesions (PI‐RADS 5), detection rates declined with increasing prostate volume, indicating that prostate size impacts biopsy performance independently of imaging suspicion.

Several mechanisms may explain this finding. Larger prostates may dilute tumor burden relative to total gland volume, reduce sampling efficiency, and increase spatial mismatch during MRI–US fusion [35, 37]. In addition, the observed shift toward anterior lesion localization in larger glands may further impair detection due to reduced accessibility.

### 4.5. Comparison With Existing Literature

Our results are consistent with the majority of published studies demonstrating an inverse relationship between prostate volume and cancer detection [32, 38, 42, 46, 53–60].

For example, Ung et al. reported decreasing detection rates with increasing prostate size (40% vs. 24%) [32], while Yoon et al. described a more moderate decline (30% vs. 26.9%) [38]. Özden et al. observed a marked decrease in csPCa detection from 61.5% in prostates < 30 mL to 6.9% in prostates ≥ 80 mL [53]. Similarly, Karakiewicz et al. demonstrated a strong inverse association in a large cohort [54].

Alternative imaging‐based approaches have also been explored. Colleselli et al. showed improved detection using CEUS in smaller prostates [46], while Al‐Azab identified small prostate volume as a key predictor in patients with PSA levels between 2.0 and 9.0 ng/mL [55].

In contrast, Diaz et al. reported higher detection rates in larger prostates [56], and Uzzo et al. suggested that biopsy strategy may have a stronger impact than gland size alone [57]. Chen et al. further highlighted that small tumor foci may be more prevalent in larger prostates, requiring more extensive sampling [58].

Our study extends these findings by demonstrating that the negative impact of prostate volume persists even in MRI‐targeted biopsy settings and across PI‐RADS strata.

### 4.6. Tumor Aggressiveness and Prostate Volume

With regard to tumor biology, our analysis did not demonstrate a consistent relationship between prostate volume and tumor aggressiveness. Although a higher proportion of high‐risk cancers was observed in the subgroup with prostate volumes of 80–119.9 mL, no linear trend across all volume groups was identified.

This finding suggests that prostate size primarily affects detection probability rather than intrinsic tumor aggressiveness. In other words, larger glands do not necessarily harbor less aggressive disease, but clinically significant tumors may be more difficult to detect.

These observations are in line with Gershman et al., who reported an association between smaller prostate weight and high‐grade disease as well as Gleason score upgrading [59]. Similarly, Yilmaz et al. described an association between smaller prostate volume and high‐grade cancer, although with only moderate predictive accuracy (AUC: 0.63) [60].

### 4.7. Clinical Implications

These findings have important clinical implications. Our data suggest that the diagnostic utility of DRE decreases with increasing prostate volume. Although biopsy positivity among DRE‐positive patients remained high, the proportion of patients with a positive DRE declined markedly in larger glands, indicating reduced sensitivity in this subgroup.

First, prostate volume should be considered when interpreting negative biopsy results, particularly in patients with large glands. Second, biopsy strategies may need to be adapted in this population, for example, by increasing sampling density or using transperineal approaches.

Finally, the reduced diagnostic performance observed in large prostates highlights the need for further technical refinement of MRI–US fusion techniques and improved targeting strategies.

### 4.8. Study Limitations

This study has several limitations. Its retrospective design introduces the potential for selection bias. In addition, variability in MRI acquisition and biopsy technique across centers may have influenced the results, although all examinations were performed according to standardized PI‐RADS protocols.

Furthermore, lesion‐level correlation between imaging findings and histopathology was not available, limiting the ability to differentiate between in‐lesion and out‐of‐lesion cancer detection.

Finally, subgroup analyses—particularly in very large prostates—were based on relatively small sample sizes and should therefore be interpreted with caution.

## 5. Conclusion

Prostate volume is a key and independent determinant of PCa detection in MRI–US fusion‐guided biopsy. Our findings demonstrate a consistent inverse relationship between gland size and diagnostic yield, with markedly reduced detection rates in larger prostates—even in the presence of highly suspicious MRI findings. These results highlight that prostate volume is not merely a descriptive parameter but a clinically relevant factor that directly impacts biopsy performance. Accordingly, prostate size should be systematically integrated into the diagnostic pathway alongside PSA, DRE, and PI‐RADS assessment. Incorporating prostate volume into clinical decision‐making may improve risk stratification, interpretation of negative biopsy results, and selection of appropriate biopsy strategies, particularly in patients with enlarged glands. Future prospective studies are warranted to refine volume‐adapted biopsy approaches and to further optimize diagnostic accuracy in this challenging patient population.

## Author Contributions

Conception and design: Toni Franz; data acquisition: Toni Franz and Tom Sicker; data analysis and interpretation: Toni Franz; statistical analysis: Toni Franz; figure design: Toni Franz and Tom Sicker; manuscript drafting: Toni Franz and Tom Sicker; critical revision of the manuscript: Julian Lueke, Benny Dinh, Christoph Breininger, and Tom Sicker; and supervision: Toni Franz, Evangelos Liatsikos, and Jens‐Uwe Stolzenburg.

## Funding

Open‐access funding was enabled and organized by Projekt DEAL.

All authors declare that the development of the manuscript was not supported by an honorarium, a grant, or any other sources of support, including sponsorship or any material sources of support from any organization for the submitted work.

## Disclosure

All authors have declared that there are no other relationships or activities that could appear to have influenced the submitted work.

## Ethics Statement

The submitted manuscript meets all criteria of the ICMJE recommendations and COPE guidelines. The requirement for ethical approval was waived by the Ethics Committee at the Medical Faculty of the University of Leipzig. Reference number: 280/25‐ek. “There are no ethical and scientific concerns.” All procedures performed in this study have been performed in accordance with the ethical standards laid down in the 1964 Declaration of Helsinki and its later amendments or comparable ethical standards of the institutional and national research committee.

## Consent

Informed consent was obtained from all individual participants included in the study.

## Conflicts of Interest

The authors declare no conflicts of interest.

## Data Availability

The data that support the findings of this study are available on request from the corresponding author.
